# Vortioxetine Prevents Lipopolysaccharide-Induced Memory Impairment Without Inhibiting the Initial Inflammatory Cascade

**DOI:** 10.3389/fphar.2020.603979

**Published:** 2021-02-04

**Authors:** S. Alboni, C. Benatti, C. Colliva, G. Radighieri, J. M. C. Blom, N. Brunello, F. Tascedda

**Affiliations:** ^1^ Department of Life Sciences, University of Modena and Reggio Emilia, Modena, Italy; ^2^ Dept. of Biomedical, Metabolic and Neural Sciences, University of Modena and Reggio Emilia, Modena, Italy; ^3^ Centre of Neuroscience and Neurotechnology, University of Modena and Reggio Emilia, Modena, Italy; ^4^ CIB, Consorzio Interuniversitario Biotecnologie, Trieste, Italy

**Keywords:** microglia, sickness behavior, lipopolysaccharide (LPS), vortioxetine, hippocampus, neuroinflammation, memory, cognition

## Abstract

Vortioxetine is a novel multimodal antidepressant that modulates a wide range of neurotransmitters throughout the brain. Preclinical and clinical studies have shown that vortioxetine exerts positive effects on different cognitive domains and neuroprotective effects. Considering the key role of microglial cells in brain plasticity and cognition, we aimed at investigating the effects of pretreatment with vortioxetine in modulating behavioral and molecular effects induced by an immune challenge: peripheral injection of lipopolysaccharide (LPS). To this purpose, C57BL/6J male mice were first exposed to a 28-day standard diet or vortioxetine-enriched diet, which was followed by an acute immune challenge with LPS. Sickness symptoms and depressive-like behaviors (anhedonia and memory impairment) were tested 6 and 24 h after exposure to LPS, respectively. Moreover, the expressions of markers of immune activation and M1/M2 markers of microglia polarization were measured in the dorsal and ventral parts of the hippocampus. The pretreatment with vortioxetine did not affect both LPS-induced sickness behavior and anhedonia but prevented the deficit in the recognition memory induced by the immune challenge. At the transcriptional level, chronic exposure to vortioxetine did not prevent LPS-induced upregulation of proinflammatory cytokines 6 h after the immune challenge but rather seemed to potentiate the immune response to the challenge also by affecting the levels of expression of markers of microglia M1 phenotype, like cluster of differentiation (CD)14 and CD86, in an area-dependent manner. However, at the same time point, LPS injection significantly increased the expression of the M2 polarization inducer, interleukin 4, only in the hippocampus of animals chronically exposed to vortioxetine. These results demonstrate that a chronic administration of vortioxetine specifically prevents LPS-induced memory impairment, without affecting acute sickness behavior and anhedonia, and suggest that hippocampal microglia may represent a cellular target of this novel antidepressant medication. Moreover, we provide a useful model to further explore the molecular mechanisms specifically underlying cognitive impairments following an immune challenge.

## Introduction

One of the main aims of neuropharmacological research is to develop more effective strategies for treating major depressive disorder (MDD). In fact, while the impact of this disease on society is impressive and continuously rising, current treatments for MDD are still quite inadequate, making the management of this psychiatric disorder a clinical challenge (Herrman et al., 2019).

Basic research is fundamental to enhance our understanding of the molecular and cellular mechanisms involved in the development of MDD. Because symptoms of depression range from neurovegetative signs to impaired mood and cognition, research efforts should be directed to distinguish common molecular patterns from specific ones, which participate in determining both differences in response to treatment and the underlying pathophysiology of MDD (Fox and Lobo, 2019).

An increasing number of studies demonstrate that, in MDD, the equilibrium between health and disease, depends on the complex balance between the nervous, endocrine, and immune systems (Alboni et al., 2013b; Furtado and Katzman, 2015; Horowitz and Zunszain, 2015; Benatti et al., 2016). Intrinsic and extrinsic factors participate in determining the status of this dynamic equilibrium (Lund et al., 2018). For instance, lipopolysaccharide (LPS), the endotoxin derived from Gram-negative bacteria, induces different behavioral changes by perturbating this integrated system. In fact, the peripheral administration of LPS in animals activates the hypothalamic-pituitary-adrenal (HPA) axis and the immune response and affects behavior (Dantzer, 2004). LPS-induced effects on behavior include transient early symptoms of sickness, such as the reduction of food intake and explorative activity and depressive-like behaviors, including reduced motivation, anhedonia, and cognitive impairments (Konsman et al., 2002; Dantzer et al., 2008). Similarly, in humans, LPS administration induces anxiety, depressed mood, and cognitive impairments (Kullmann et al., 2013).

Therefore, peripheral administration of LPS represents a useful tool to study the molecular and cellular mechanisms underlying different, adaptive and/or maladaptive, behavioral responses to an immune challenge perturbating the neuro-immune-endocrine system (Wu et al., 2016; Rodrigues et al., 2018).

Different studies showed that pretreatment with different molecules prevents LPS-induced changes in behavior (Henry et al., 2008; O’Connor et al., 2009b; Basu Mallik et al., 2016; Shi et al., 2017). These studies suggest that molecules reducing LPS-stimulated cytokines induction in the brain partially prevent behavioral sequelae as well (Ferreira Mello et al., 2013; Kobayashi et al., 2013; Jangra et al., 2014; Sulakhiya et al., 2014, Sulakhiya et al., 2016; Shi et al., 2017).

Vortioxetine (VTX) is a novel antidepressant that has proven particularly effective in treating cognitive impairments associated with psychiatric diseases (McIntyre et al., 2014; Frampton, 2016; Salagre et al., 2018; Bruno et al., 2020). VTX-induced effects on memory and cognition seem to depend on its unique pharmacologic profile. In fact, VTX is a multimodal antidepressant drug able to directly or indirectly modulate a wide range of neurotransmitters (i.e., serotonin, dopamine, norepinephrine, histamine, glutamate, and GABA) by acting on several targets (i.e., partial agonism at 5-HT1B and agonism at 5-HT1A receptors and antagonist properties at 5-HT1D, 5-HT3, and 5-HT7 receptors) (Schatzberg et al., 2014; Sanchez et al., 2015). VTX exhibits neuroprotective effects and prevents cognitive impairments in different animal models of depression (du Jardin et al., 2014; Jensen et al., 2014; Wallace et al., 2014; Smith et al., 2018; Torrisi et al., 2019; Jiang et al., 2020).

The main central target of VTX appears to be neurons (Bétry et al., 2015; Chen et al., 2016; Waller et al., 2016). However, immunomodulatory properties for VTX have recently been described on human monocytes/macrophages (Talmon et al., 2018). Given the role of microglia cells, the brain innate immune cells, in brain plasticity and thus cognition (Branchi et al., 2014; Alboni and Maggi, 2016), it is reasonable to assume that VTX, directly or indirectly, may affect microglia functionality. Recently, Tomaz et al. explored the effect of different antidepressants, including VTX, in preventing LPS-induced effects in rats (Tomaz et al., 2020). However, most studies exploring the behavioral and molecular effects induced by LPS exposure employ mice, and no evidence so far has been found that VTX is able to prevent LPS-induced effects on cognition.

Given the above, we tested the ability of VTX to modulate LPS-induced effects on different behavioral and molecular alterations following the immune challenge. In particular, we evaluated early induced sickness symptoms and subsequent effects on motivation (anhedonia) and memory. Transcriptional effects on markers of immune activation and microglia phenotype were assessed 6 h after LPS injection in the dorsal and ventral parts of the hippocampus. Even though the response to systemic stressors involves an extensive circuitry including many structures of the limbic forebrain, the hippocampal formation represented our main focus since it has major control over the HPA axis and leads pivotal integrative processes over endocrine and inflammatory cues that set the motor, behavioral, and cognitive impairments that occur during a systemic inflammatory response. Our choice to focus on the dorsal and ventral hippocampi was based on the currently accepted view that these areas are morphologically and functionally different (Moser and Moser, 1998; Bartsch and Wulff, 2015; Floriou-Servou et al., 2018), and that the systemic administration of LPS was proven to affect their functional state and the endocrine response in a region-dependent manner (Fanselow and Dong, 2010; Onufriev et al., 2018).

## Materials and Methods

### Animals

Adult C57BL/6J male mice (*n* = 87; Charles River Laboratories, Lecco, Italy) between 11 and 16 weeks of age were used in all experiments. Mice were habituated for 5 weeks prior to pharmacological treatment and group-housed (four per cage) in polycarbonate cages (30 × 30 × 15 cm) with *ad libitum* access to food and water throughout the study while being maintained under a 12/12 h light-dark cycle (lights on 8:00 am to 8:00 pm), in an ambient temperature of 21 ± 3 °C with relative controlled humidity. Animals were checked daily for signs of discomfort as indicated by the animal care and use guidelines [National Academy of Sciences. Guide for the Care and Use of Laboratory Animals, 1998, “Guidelines for the Care and Use of Mammals in Neuroscience and Behavioral Research” (National Research Council 2003)]. All procedures were carried out in accordance with the EC guidelines (EEC Council Directive 86/609 1987) and the Italian legislation on animal experimentation (Decreto Legislativo 26/2014) and had the approval of the local Ethical Committee. Mice were handled twice a week throughout the experiment to habituate the animals to the experimenters.

### Treatment

The VTX group was fed Purina 5001 rodent chow containing VTX (synthesized by H. Lundbeck A/S, Valby, Denmark) at a concentration of 600 mg base per kg food for 28 days (Research Diets Inc., New Brunswick, NJ) and had *ad libitum* access to plain tap water. The control group was fed Purina 5001 rodent chow with continuous access to plain tap water. Animals were weighed once a week, and food and water intake of each cage was assessed twice a week: no gross changes in animal’s feeding and drinking behavior were found (Supplementary result and Supplementary Figure S1). The concentration of VTX was chosen to reach the therapeutic dose range based on brain SERT occupancy resulting from previous studies: mice fed with the medicated diet ingested a mean of 2.84 mg/die of VTX base according to their daily food intake (VTX: 4.73 ± 0.45 g/die/mice; Ctrl 4.75 ± 0.59 g/die/mice; Mean ± SD) (Li et al., 2015).

**FIGURE 1:**
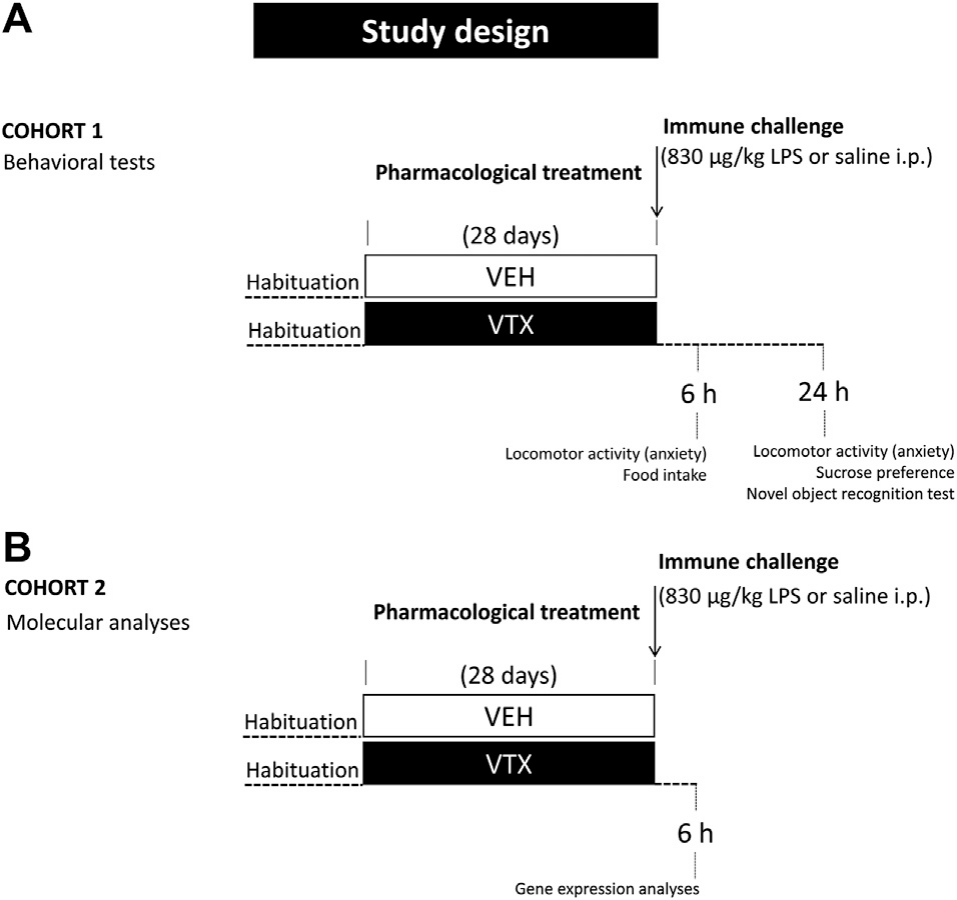
Experimental design: after habituation, animals were fed for 28 days with standard diet or vortioxetine-enriched diet (600 mg base per kg of food) and then intraperitoneally injected with lipopolysaccharide (LPS 830 μg/kg) or an equal volume of vehicle (pyrogen-free saline). **(A)** A group of animals (cohort 1; *n* = 48) was tested 6 and 24 h after the immune challenge for LPS-induced behaviors and depressive-like behavior, respectively. **(B)** An independent group of animals (cohort 2; *n* = 39) was sacrificed 6 h after LPS or saline injection for molecular analyses.

On the 29th day, mice were injected intraperitoneally (i.p.) with LPS from *Escherichia coli* (serotype 0127:B8), purchased from Sigma-Aldrich (St. Louis, MO, United States), at a dose of 830 μg/kg (*n* = 44) or vehicle (pyrogen-free saline) (*n* = 43). For habituation to the treatment procedure, mice were handled 2 days before the injection. On the day of the treatment, animals were weighed before and 6 h after LPS or saline administration to evaluate mean body weight. Food was also weighed for the evaluation of treatment-induced effects on feeding. Animals were divided into two cohorts for the assessment of behavioral (cohort 1; *n* = 48) and molecular (cohort two; *n* = 39) LPS-induced effects (Figure 1).

### Behavioral Assessment

Animals belonging to cohort 1 were checked 6 h after LPS or vehicle injection for a sickness-induced response (feeding and body weight loss, symptoms of sickness, and behavior in the open field test) and 24 h after the immune challenge for depressive-like behaviors. In particular, we evaluated anhedonia using the sucrose preference test and cognitive impairment with the novel object recognition test. The total amount of liquid intake during the sucrose preference test and the open field test were also evaluated at the later time point to exclude bias possibly affecting the evaluation of the depressive-like phenotypes (Biesmans et al., 2016). Each animal underwent behavioral tests subsequently (open field, sucrose preference, and novel object recognition test) during the light cycle phase and was evaluated and analyzed by an observer blinded to the mice treatment.

#### Open Field Test

Exploratory behavior was measured in an open field box constructed of white Plexiglas (45 × 30 × 25 cm) as previously described (Benatti et al., 2011). Tests were recorded using a video camera. Animals were accustomed to the experimental room for 1 h prior to the experiment. The floor and walls of the arena were wiped between trials, first with water and then with 30% v/v alcohol: distilled water solution to prevent mice from following the scent of a previously tested animal. The illumination in the center of the arena was 100 lux. At the beginning of the test, each mouse was placed individually in the center of the arena. In each 5 min session, the following parameters were evaluated using Smart software (version 2.5): total distance traveled and distance traveled in the central part (12 × 12 cm) of the arena (centimeters), time spent in the center, and resting time in the whole arena and the central area (seconds). Total distance traveled is an index of general locomotor (horizontal) behavior, whereas the total number of rearings is an index of locomotor activity (vertical). Time spent within the central area vs. the rest of the open field arena is a measure of anxiety-like behavior.

#### Evaluation of Sickness Behavior

Behavioral data were collected while mice were in their home cage to avoid stress associated with an unfamiliar environment. Six and 24 h after saline or LPS injection, the presence or absence of the following symptoms was evaluated: 1) curled body posture, 2) ptosis, and 3) piloerection.

#### Sucrose Preference

Anhedonia, defined as a decreased sensation of pleasure, represents a key symptom of MDD and can be assessed in mice by measuring the preference for a palatable solution (Alboni et al., 2017; Liu et al., 2018). The sucrose preference test consisted of a 3-day training and a test phase performed in the 24 h period following LPS injection, when LPS is known to induce a decrease in sucrose preference. Over the dark phase of the light cycle, single-housed mice were trained with two identical bottles containing either a freshly prepared 1% sucrose solution or water. To control for a side preference in drinking behavior, the position of the two identical bottles was switched every day, and to reduce neophobia in the week before, the test animals were habituated to drink from two identical bottles filled with water in their home cage. Prior to and during testing, mice were not food- and water-deprived. Fluid consumption (grams) was measured by weighing bottles before and after each session. Sucrose preference was calculated as the percentage of sucrose intake volume over the total volume of fluid intake in a 24 h period (Liu et al., 2018).

#### Novel Object Recognition Test

The novel object recognition (NOR) test has emerged as the most popular test for assessing a rodent’s ability to recognize a previously presented stimulus and evaluate nonspatial memory (Antunes and Biala, 2012; Lueptow, 2017). To test a discrete form of learning and memory that involves the hippocampal formation, animals were tested in boxes from the open field task. After 5 min of open field, we considered animals habituated to the arena. Thus, each animal was exposed for another 5 min to a pair of objects that differed in shape, surface color, contrast, and texture (training session). Three hours and 30 min after the initial exposure, mice were reexposed, in the same arena, for 8 min to one of the original objects and a novel object (test session). The objects were previously shown to induce approximately equal time of exploration in C57BL6/J and were located 10 cm from the side walls. These objects were randomized so that each object could be used either as a familiar or novel object in any given session (Lueptow, 2017). In all tests, the time spent exploring each object was recorded. A mouse was considered to engage in exploratory behavior if it touched the object with its forepaw or nose or sniffed the object within a distance of 1.5 cm. After every exposure, the objects and the cage were wiped with water and then with 30% v/v alcohol: distilled water solution to eliminate odor cues.

### Molecular Analyses

Six hours after LPS or saline injection, animals belonging to cohort 2 were sacrificed, trunk blood was collected, brains were removed, and ventral and dorsal hippocampi were dissected and immediately stored at −80 °C until RNA extraction. This time point was selected based on published and preliminary results suggesting that changes in the expression levels of inflammatory markers at this time are indicative of the early immune response and may underlie late induced effects on behavior (Terrando et al., 2010; Biesmans et al., 2013).

#### Serum Preparation

Trunk blood was collected after decapitation for the determination of glucocorticoid levels as an indication of HPA activation. Experiments were performed from 9:00 am to 4:00 pm during the light period. To improve serum separation from whole blood, samples were let to clot 15 min at room temperature and 1 h on ice before centrifugation (1,000 × g for 15 min). Serum was transferred into clean tubes and stored at −80 °C until the assay.

#### Corticosterone Serum Levels

Assessment of serum corticosterone levels was done by means of enzyme immunoassay (EIA) using a commercially available kit (Arbor Assays, Ann Arbor, MI, United States), which utilizes a microplate reader set at 450 nm, following the manufacturer’s instructions. Serum samples were diluted 1:150 in appropriate assay buffer and assayed in duplicate. The detection limit of the assay was 16.9 pg/ml; intra- and interassay coefficients of variations were 8.15% and 17.93%, respectively.

#### Total RNA Extraction, Reverse Transcription, and Real-Time Polymerase Chain Reaction

RNA extraction and DNAse treatment were performed as previously described (Alboni et al., 2013a) using GenElute™ Mammalian Total RNA Miniprep Kit and DNase70-On-Column DNAse I Digestion Set (Sigma-Aldrich®, Milan, Italy). Two µg of total RNA was reverse-transcribed with High Capacity cDNA Reverse Transcription Kit (Thermo Fisher Scientific, Waltham, MA, United States) and Real-Time PCR was performed, as previously described (Benatti et al., 2011), in ABI PRISM 7900 HT (Thermo Fisher Scientific, Waltham, MA, United States) using Power SYBR Green mix (Thermo Fisher Scientific, Waltham, MA, United States) and specific forward and reverse primers at a final concentration of 150 nM (see Supplementary Table S1 for primer sequences). Ct (cycle threshold) value was determined by the SDS software 2.2.2 (Thermo Fisher Scientific, Waltham, MA, United States); mRNA expression was calculated with the ΔΔCt method with cyclophilin A (CypA) as endogenous control.

### Statistical Analysis

All statistical analyses were performed using SPSS software ver. 26.0 (IBM Corp., Armonk, NY, United States). Extreme outliers were excluded prior to statistical analysis using the boxplot tool in SPSS (more than 3x the interquartile range outside of the end of the interquartile box). Molecular and behavioral data were analyzed with two-way analysis of variance (ANOVAs) for main effects of LPS exposure, diet, or interaction between the two factors (LPS/saline * standard/VTX-enriched diet). When a significant main effect was found, planned pairwise comparisons were performed by one-way ANOVA followed by Tukey’s HDS p*ost hoc* test (LPS *vs.* saline animals receiving the same diet for 4 weeks or standard *vs.* VTX-enriched diet in mice injected with either LPS or saline). Analysis of NORTs involved one-sample *t*-tests comparing each group’s mean percent preference with a hypothetical mean of 50% chance preference (Keppel 1991). A *p* level of <0.05 was regarded as significant for all tests.

## Results

### Chronic Pretreatment With VTX Does Not Prevent LPS-Induced Sickness Behavior 6 h after the Immune Challenge

**FIGURE 2:**
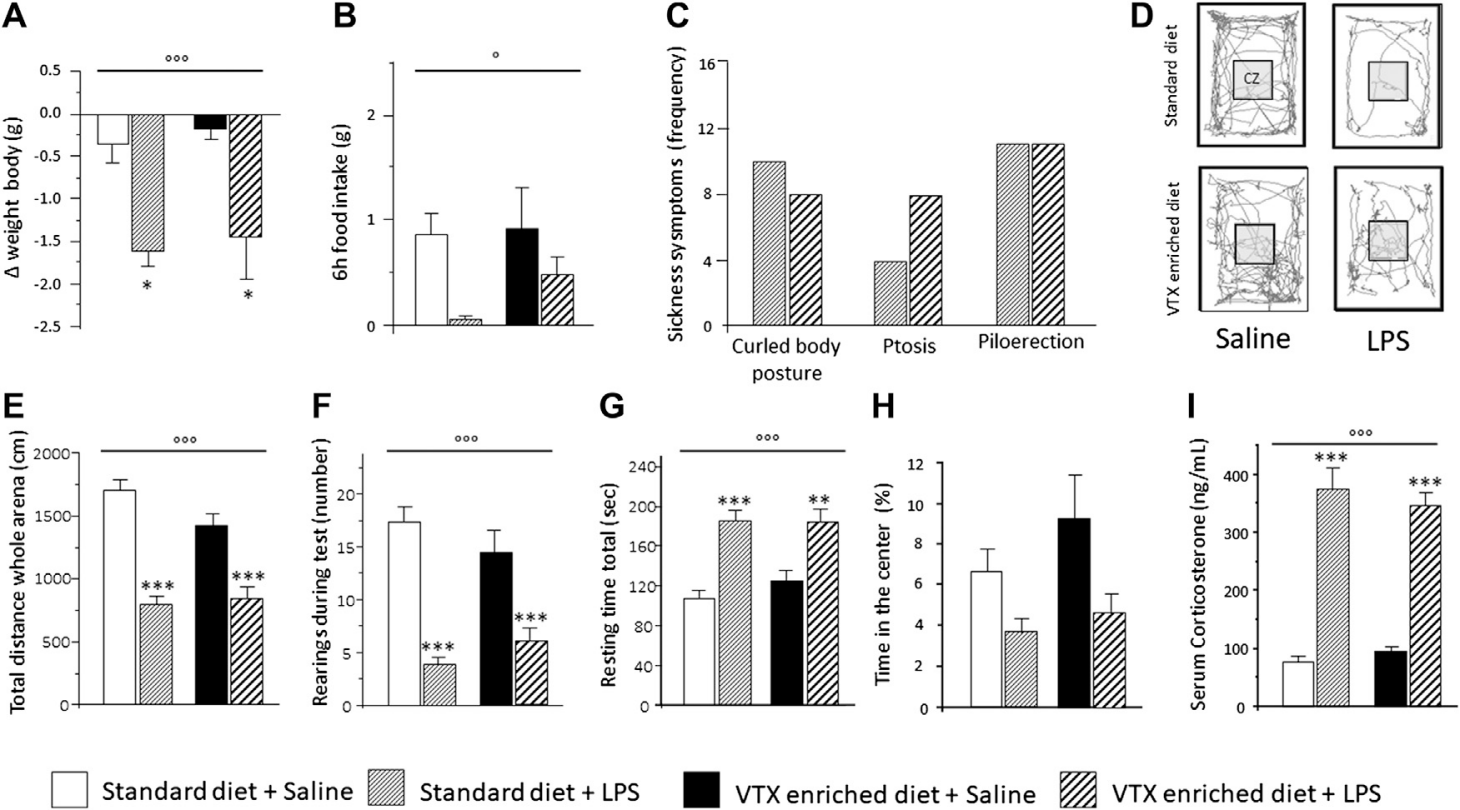
Six hours after LPS injection, the physiological and behavioral effects related to the sickness did not differ between mice that received standard diet as compared to mice that received VTX-enriched diet before the immune challenge. Histograms **(A–C)** represent **(A)** the variation in body weight and **(B)** food intake and **(C)** the frequency of symptoms of sickness behavior in LPS-injected animals previously fed with standard diet or VTX-enriched diet. **(D)** Representative movement traces of all groups in the open field. CZ, central zone. Histograms **(E–H)** represent the behaviors in the open field test; **(E)** total distance moved (cm) in the entire open field area; **(F)** number of rearings during test; **(G)** resting time during test; **(H)** percentange of time spent in the center of the open field. **(I)** Serum corticosterone levels of animals that did not perform behavioral tests (cohort 2) 6 h after the immune challenge. Treatment as indicated in the legend, *n* = 12 mice per group except for the evaluation of frequency of symptoms of sickness behavior *n* = 18 per group. Data are represented as means ± SEM and were analyzed with ANOVA followed by Tukey’s HSD (two-way ANOVA: ^◦^ *p* < 0.05, ^◦◦◦^ *p* < 0.001 main effect LPS/saline; *post hoc*: *p < 0.05, **p < 0.01, ***p < 0.001, indicating a significant difference compared to saline-treated matching group).

The hypothesis was tested that chronic pretreatment with VTX may affect LPS-induced sickness behavior (see Supplementary Table S2 for a complete summary of statistical results). On the day of the immune challenge, before receiving either LPS or saline, no significant difference was found in body weight and food consumption between mice treated chronically with VTX and those fed with a standard diet (Figures 2A,B). A significant main effect was observed for LPS, which indicated that body weight and food intake were diminished 6 h after exposure to LPS [F (1,43) = 18.683; *p* < 0.0001 and F (1,43) = 6.687; *p* = 0.013, respectively]. *Post hoc* analysis revealed that LPS-treated mice displayed a significant reduction in weight compared to saline-injected mice, and saline-receiving animals consumed more food than their LPS-injected counterparts (Figures 2A,B). As shown in Figure 2C, after LPS injection, the frequency of symptoms of sickness did not differ between animals receiving either a standard diet or an enriched VTX diet (Figure 2C). Mice not receiving the immune challenge did not show symptoms of sickness. Six h after exposure to LPS, locomotor activity was affected. LPS had main effects on the total distance traveled (horizontal locomotor activity) and the number of rearings (vertical locomotor activity) [F (1,41) = 62.805; *p* < 0.0001 and F (1,41) = 47.596; *p* < 0.0001, respectively] and treatment with LPS reduced both these parameters, while significantly increasing total resting time [F (1,43) = 34.124; *p* < 0.0001] regardless of the diet animals received (Figures 2E–G). An increase, which failed to reach statistical significance, in anxiety-like behaviors was observed in both VTX- and standard-fed animals exposed to LPS [F (1,41) = 3.330; *p* = 0.077] (Figure 2H). Finally, we measured serum corticosterone levels, indicative of HPA axis activation, in mice belonging to cohort 2 that were not tested for behavior but instead sacrificed 6 h after LPS or saline injection. As shown in Figure 2I, serum corticosterone levels were increased after LPS treatment in both animals receiving standard or VTX-enriched diet [F (1,42) = 153.023; *p* < 0.0001] (Figure 2I).

Together, these results confirmed the well-known capability of LPS to induce various types of sickness-related behaviors and demonstrated that chronic pretreatment with VTX does not alter the ability of the immune challenge to affect these behavioral parameters 6 h after exposure.

### Chronic Pretreatment with VTX Specifically Prevents LPS-Induced Effects on Memory 24 h after the Immune Challenge

Twenty-four hours after LPS/saline injection, we assessed the depressive-like phenotypes in animals that were fed for 28 days either with a standard or VTX-enriched diet before receiving the immune challenge (see Supplementary Table S2 for a complete summary of statistical results). At this later time point, sickness is expected to be minimal and will not bias the measurement of depressive-like behaviors.

**FIGURE 3:**
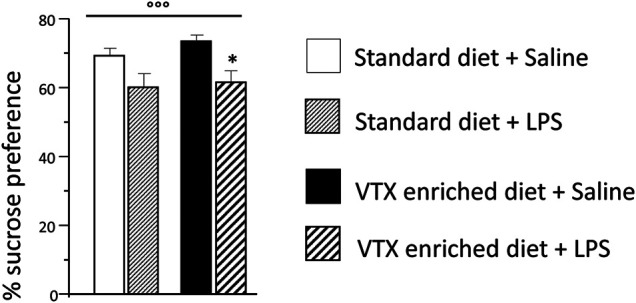
The effect of an immune challenge on sucrose preference (test of anhedonia/motivation) was similar in animal receiving either standard or VTX-enriched diet for 4 weeks. Histogram representing the sucrose preference (percentage of the volume of sucrose intake over the total volume of liquid intake) measured for a 24 h period starting at the time of LPS/saline injection. Treatment as indicated in the legend, *n* = 12 mice per group. Data are represented as means ± SEM and were analyzed with ANOVA followed by Tukey’s HSD (two-way ANOVA: ^◦◦◦^ *p* < 0.001 main effect LPS/saline; *post hoc*: *p < 0.05 is a significant difference compared to saline-treated matching group).

LPS significantly reduced sucrose preference [F (1,37) = 12.718; *p* = 0.001], whereas no main effect was observed for treatment. Animals injected with LPS showed a lower preference for a 1% sucrose solution compared to saline-injected animals (Figure 3). The total amount (sucrose solution plus water) of liquid intake did not differ among the experimental groups, suggesting that sucrose preference depends on different motivational behavior and not on an unspecific effect related to liquid intake (Supplementary Table S2).

LPS-induced effects on recognition memory were evaluated with the NOR Test (NORT). Before the beginning of the test, the explorative behavior was evaluated for 5 min in the open field apparatus to verify that the alterations observed 6 h after LPS injection were no longer present at the time of the memory test and ruled out any confounding effect on the results (see Supplementary Table S2 for a complete summary of statistical results). Twenty-four hours after LPS/saline injection, none of the behavioral alterations observed 6 h after injection was still present (Supplementary Figure S2).

**FIGURE 4:**
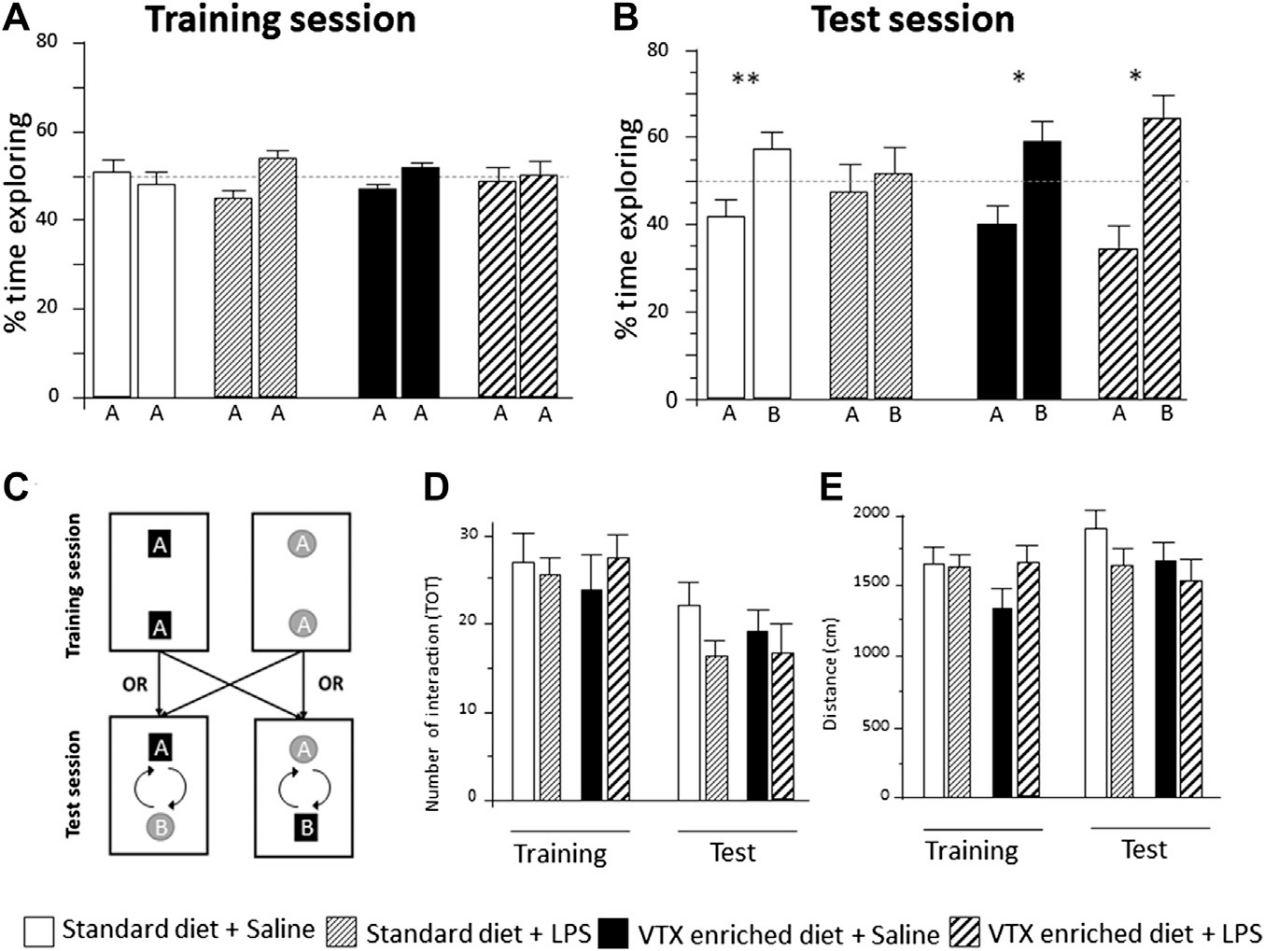
In mice receiving chronic treatment with VTX, LPS did not disrupt learning, 24 h after endotoxin injection. Histograms represent **(A)** % time exploring objects during the training session, experimental groups did not differ before the immune challenge; **(B)** animals’ exploration preference between the familiar and the novel object in a memory retention test (percent of time exploring each object over total), A = the familiar object and B = the novel object; **(C)** schematic representation of the arena setup used in the object recognition. During the training session, two identical objects were placed in the apparatus. The test trial takes place 3.5 h later with the placement of a novel object. **(D)** The number of total interactions with the objects during training and test session; **(E)** distance traveled in the NORT box during training and test sessions. Treatment as indicated in the legend, *n* = 12 mice per group. Data are represented as mean ± SEM and were analyzed with one-sample *t*-test (*p < 0.05; **p < 0.01 indicate a significant difference with a mean of 50% chance preference).

All groups behaved similarly during the training session: they spent about 50% of the allotted time exploring each object (Figure 4A and Supplementary Table S2). VTX pretreatment inhibited LPS-induced cognitive impairment in the test session. Saline-injected animals fed with a standard diet spent significantly more time (∼60%) exploring the novel object vs. the familiar one (one-sample *t*-test novel vs. 50% chance preference: *t* = −5.474, *p* = 0.001), whereas their LPS-exposed counterparts showed no preference, exploring for half of the time each object, suggesting an inability to remember the familiar object (*t* = −0.340, *p* = 0.740). When treated with VTX for 4 weeks, both saline- and LPS-exposed mice spent a significantly higher amount of time exploring the novel object compared to the familiar one (*t* = −3.053, *p* = 0.014; *t* = −2.866, *p* = 0.015, respectively) (Figure 4B). In the test session, the total number of interactions with the objects (Figure 4D), total distance traveled (Figure 4E), and total time exploring the objects (Supplementary Table S2) did not differ between groups.

### Hippocampal Expression of Inflammatory Markers Following Exposure to LPS: Effect of VTX Pretreatment

We then assessed the effects of chronic VTX pretreatment on LPS-induced changes at the molecular level, measuring the hippocampal gene expression of immune markers 6 h after LPS or saline injection (see Supplementary Table S3 for a complete summary of statistical results).

#### LPS-Induced Expression of Proinflammatory Cytokines Is Not Inhibited by VTX Chronic Pretreatment

**FIGURE 5:**
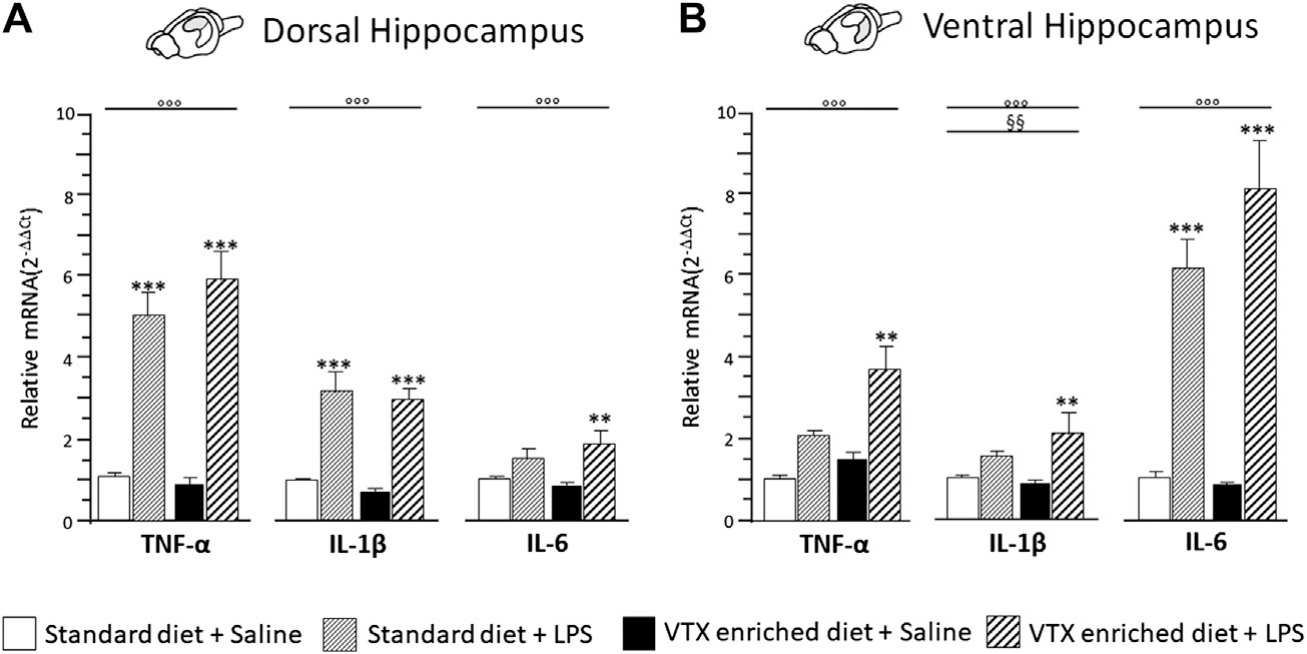
The effect of an immune challenge on the expression of proinflammatory cytokines in the dorsal and ventral hippocampus was similar in animals chronically fed with a standard diet or VTX-enriched diet. Gene expression results are shown as fold changes relative to saline-treated control (standard diet) group. Bar graphs representing the expression of selected proinflammatory cytokines (TNF-α, IL-1β, and IL-6) in the **(A)** dorsal hippocampus or in the **(B)** ventral hippocampus. Treatment as indicated in the legend, *n* = 9–10 mice per group. Data are represented as means ± SEM and were analyzed with ANOVA followed by Tukey’s HSD (two-way ANOVA: ^◦◦◦^ *p* < 0.001 main effect LPS/saline; ^§§^ *p* < 0.01 main effect standard/VTX-enriched diet; *post hoc* **p < 0.01, ***p < 0.001 indicate significant difference compared to saline-treated matching group).

A two-way ANOVA revealed a significant main effect of LPS for all the cytokines evaluated in both the dorsal hippocampus [TNF-α: F (1,36) = 96.822, *p* < 0.0001; IL-1β: F (1,35) = 43.759, *p* < 0.0001; IL-6: F (1,36) = 14.046, *p* = 0.001] and the ventral hippocampus [TNF-α: F (1,37) = 88.535, *p* < 0.0001; IL-1β: F (1,36) = 23.375, *p* < 0.0001; IL-6: F (1,37) = 13.547, *p* = 0.001]. Moreover, a main effect of VTX-enriched diet was present [F (1,36) = 9.119, *p* = 0.005] but only for IL-1β expression levels in the ventral hippocampus; none of the analyses performed revealed an interaction among the main factors. *Post hoc* analysis showed that LPS significantly increased the expression of all the three cytokines in both areas of the hippocampus of mice treated chronically with VTX compared to their saline-exposed counterparts. In control animals, only TNF-α and IL-1β mRNAs expressions were strongly increased by LPS in the dorsal hippocampus, while a significant increase in mRNA coding for IL-6, but not TNF-α and IL-1β mRNAs, was observed in the ventral hippocampus of LPS-exposed animals when compared with control mice receiving saline (Figures 5A,B).

#### LPS Induced the Expression of Indoleamine 2,3-Dioxygenase in the Dorsal Hippocampus in Mice Exposed to Chronic Treatment with VTX

**FIGURE 6:**
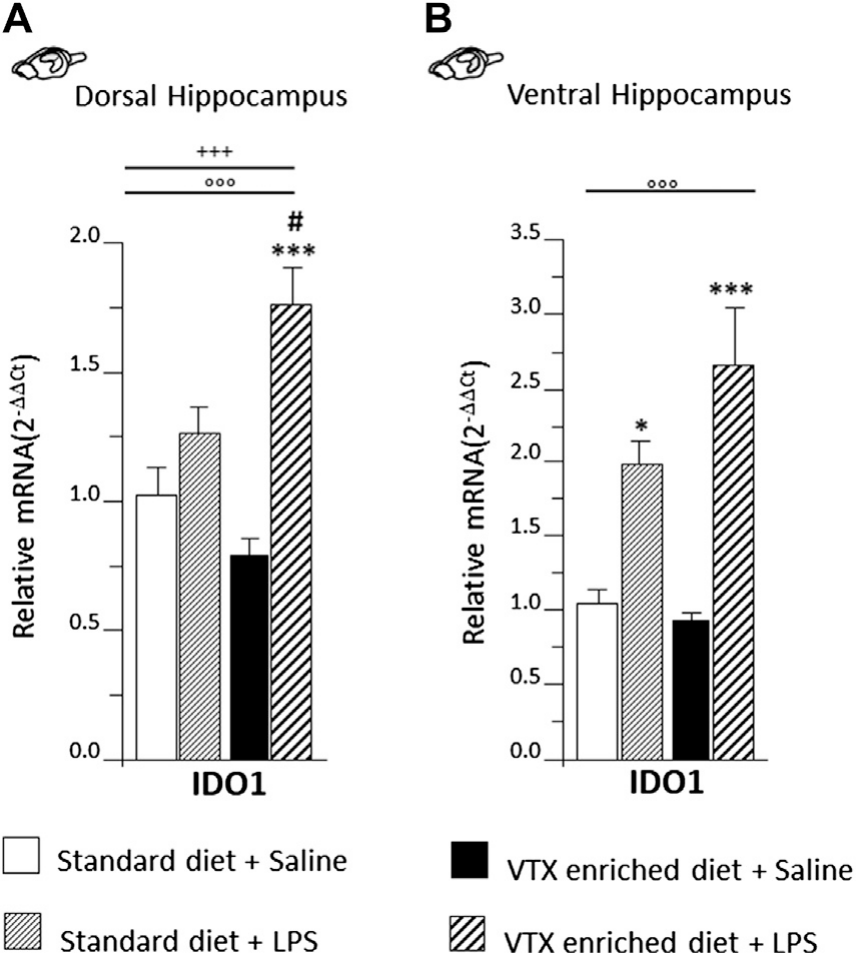
LPS injection strongly induced IDO1 expression only in the dorsal hippocampus of animals that received chronic treatment with VTX, while in the ventral hippocampus, IDO was significantly higher in LPS-treated mice regardless of treatment. Gene expression results are shown as fold changes relative to saline-treated control (standard diet) group. Bar graphs representing the expression of IDO1 in the **(A)** dorsal hippocampus or in the **(B)** ventral hippocampus. Treatment as indicated in the legend, *n* = 9–10 mice per group. Data are represented as means ± SEM and were analyzed with ANOVA followed by Tukey’s HSD (two-way ANOVA: ^◦◦◦^ *p* < 0.001 main effect LPS/saline; ^+++^ *p* < 0.001 LPS/saline* standard/VTX-enriched diet interaction; *post hoc* *p < 0.05, ***p < 0.001 indicate significant difference compared to saline-treated matching group; ^#^ *p* < 0.05 indicates significant difference compared to matching control group).

A two-way ANOVA revealed a significant main effect of LPS on indoleamine 2,3-dioxygenase (IDO1) expression both in the dorsal [F (1,36) = 33.931, *p* < 0.0001] and ventral [F (1,36) = 42.075, *p* < 0.0001] subregions of the hippocampus, but an interaction for LPS and treatment was observed only in the dorsal hippocampus [F (1,36) = 12.669; *p* = 0.001]. *Post hoc* analysis showed that in the dorsal portion of the hippocampus, IDO1 expression was increased following LPS only in the group treated chronically with VTX (Figure 6A), while in the ventral hippocampus, LPS elicited a similar effect 6 h after the immune challenge: an increase in mRNA levels of IDO1 was observed in both VTX treated and control mice exposed to LPS and not in saline-treated mice (Figure 6B).

#### LPS-Induced Effects on the Expression Levels of Markers of Microglia Proinflammatory (M1) Phenotype: Effect of VTX Pretreatment

**FIGURE 7:**
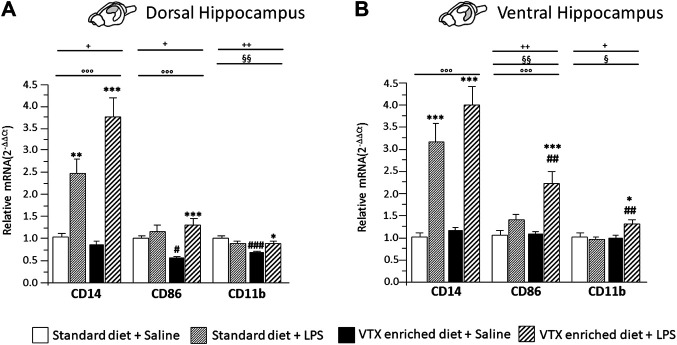
Chronic exposure to VTX enhanced the LPS-induced effects in stimulating the expression of markers of activated microglia; a negative effect of VTX on the expression of these markers suggests the involvement of an M1 phenotype in the dorsal hippocampus of saline-treated mice. Gene expression results are shown as fold changes relative to saline-treated control (standard diet) group. Bar graphs representing the expression of CD14, CD86, and CD11b mRNAs in the **(A)** dorsal hippocampus or in the **(B)** ventral hippocampus. Treatment as indicated in the legend, *n* = 9–10 mice per group. Data are represented as means ± SEM and were analyzed with ANOVA followed by Tukey’s HSD (two-way ANOVA: ^◦◦◦^ *p* < 0.001 main effect LPS/saline, ^§§^ *p* < 0.01, ^§^ *p* < 0.01 main effect standard/VTX-enriched diet; ^+^ *p* < 0.05, ^++^ *p* < 0.01 LPS/saline * standard/VTX-enriched diet interaction; *post hoc* *p < 0.05, **p < 0.01, ***p < 0.001 indicate significant difference compared to saline-treated matching group; ^#^ *p* < 0.05, ^##^ *p* < 0.01, ^###^ *p* < 0.001 indicate significant difference compared to matching control group).

A main effect for LPS treatment was observed for the expression levels of cluster of differentiation molecules (CD)14 and CD86 in both the dorsal and ventral hippocampus [for CD14 F (1,37) = 61.357, *p* < 0.0001, in dorsal and F (1,36) = 75.758, *p* < 0.0001, in ventral hippocampus; CD86 F (1,37) = 14.777, *p* < 0.0001, in dorsal and F (1,37) = 27.468, *p* < 0.0001, in ventral hippocampus]. Diet significantly affected CD86 expression in the ventral hippocampus [F (1,37) = 8.862, *p* = 0.005]. Moreover, an interaction between LPS and diet was observed for the expression of CD14 in the dorsal hippocampus [F (1,37) = 6.991, *p* = 0.012] and for CD86 in both parts of hippocampus [F (1,37) = 7.240, *p* = 0.011; F (1,37) = 7.958, *p* = 0.008 respectively]. As for CD11b, a significant main effect of diet was observed, both in the dorsal and in the ventral hippocampus [F (1,38) = 9.296; *p* = 0.004 and F (1,36) = 4.682; *p* = 0.038 respectively], as well as an interaction between LPS and diet [F (1,38) = 11.456, *p* = 0.002; F (1,36) = 7.047, *p* = 0.012, for dorsal and ventral hippocampus, respectively]. *Post hoc* analysis showed that a 4-week exposure to VTX significantly reduced CD86 and CD11b mRNA expression levels in the dorsal hippocampus of saline-treated mice compared to their counterparts receiving standard diet. In addition, CD86 and CD11b mRNAs expression was significantly enhanced by LPS in both the hippocampal subregions only in animals previously exposed to VTX. CD14 expression, instead, was significantly increased in both portions of the hippocampus after exposure to LPS irrespective of the diet (Figure 7).

#### LPS-Induced Effects on the Expression Levels of Anti-Inflammatory Cytokines and Markers of the Microglia Anti-Inflammatory (M2) Phenotype: Effect of VTX Pretreatment

The observation that a VTX pretreatment seemed to potentiate, rather than inhibit, immune response prompted us to test the hypothesis that LPS affects transcriptional events of anti-inflammatory markers in the hippocampus.

**FIGURE 8:**
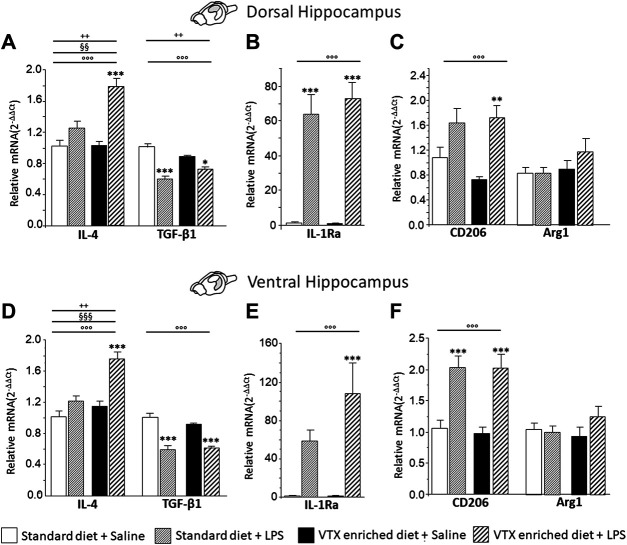
Overall, in mice-fed VTX, an immune challenge induced a more intense anti-inflammatory response in comparison to mice-fed standard chow. Gene expression results are shown as fold changes relative to saline-treated control (standard diet) group. Bar graphs representing the expression of anti-inflammatory cytokines (IL-4 and TGF-β1), cytokine antagonist (IL-1Ra), or markers of microglia M2 phenotype (CD206 and Arg1) in the dorsal hippocampus (respectively, A, B, and C) or in the ventral hippocampus (respectively, D, E, and F). Treatment as indicated in the legend, *n* = 9–10 mice per group. Data are represented as means ± SEM and were analyzed with ANOVA followed by Tukey’s HSD (two-way ANOVA: ^◦◦◦^ *p* < 0.001 main effect LPS/saline; ^§§^ *p* < 0.01, ^§§§^ *p* < 0.001 main effect standard/VTX-enriched diet, ^++^ *p* < 0.01 LPS/saline* standard/VTX-enriched diet interaction; *post hoc* *p < 0.05, ***p < 0.001 indicate significant difference compared to saline-treated matching group).

A two-way ANOVA (LPS/saline * standard/VTX-enriched diet) evidenced a significant main effect of LPS for the anti-inflammatory cytokines and IL-1Ra in both the dorsal and the ventral hippocampus (IL-4: F (1,35) = 32.073, *p* < 0.0001, and F (1,35) = 31.216, *p* < 0.0001; TGF-β1: F (1,36) = 54.109, *p* < 0.0001, and F (1,37) = 79.776, *p* < 0.0001; IL1Ra: F (1,35) = 81.799, *p* < 0.0001, and F (1,35) = 22.880, *p* < 0.0001, for dorsal and ventral hippocampus, respectively]. For IL-4, a main effect of diet and an interaction between LPS and diet were observed [standard/VTX-enriched diet: F (1,35) = 10.171, *p* = 0.003, and F (1,35) = 21.087, *p* < 0.0001; LPS * diet: F (1,35) = 9.244, *p* = 0.005, and F (1,35) = 8.493, *p* = 0.006, for dorsal and ventral hippocampus, respectively]. Moreover, for TGF-β1 mRNA expression in the dorsal hippocampus, an interaction between LPS and diet was observed [F (1,36) = 9.674, *p* = 0.004] (Figures 8A,B,D,E). The expression of CD206 was increased by LPS [F (1,37) = 19.486, *p* < 0.0001, and F (1,35) = 35.76, *p* < 0.0001, for dorsal and ventral hippocampus, respectively] irrespective of diet, while Arg1 expression tend to be higher only in animals that received a VTX-enriched diet (Figures 8C,F).

## Discussion

This study has shown that chronic administration of vortioxetine prevents LPS-induced memory impairment, without affecting acute sickness behavior, and suggests that hippocampal microglia represents a cellular target of VTX.

Acute activation of the peripheral innate immune system through the administration of the cytokine inducer LPS is commonly used model to study inflammation-associated behavioral changes in rodents (Dantzer et al., 2008; Biesmans et al., 2013). Systemic administration of LPS leads to an acute behavioral response (sickness behavior) maximal at 6 h which is followed by a depressive-like behavior, still apparent 24 h post-LPS (Konsman et al., 2002; Frenois et al., 2007; Dantzer et al., 2008; O’Connor et al., 2009b; Dantzer, 2009). Consistent with previous results, here, we found that in control animals, a systemic injection of LPS (830 μg/kg) 1) increased the expression of markers of immune activation; 2) decreased weight gain, food intake, and exploratory behavior, and activated HPA axis 6 h after the immune challenge, while 3) impairing sucrose preference and compromising memory performance in the NORT 24 h after LPS injection (Frenois et al., 2007; Gibb et al., 2008; Walker et al., 2013; Réus et al., 2015).

In our experimental conditions, a chronic treatment with VTX did not prevent either sickness behavior or the initial innate immune response induced by a single LPS peripheral injection.

Behavioral changes characteristic of sickness reflect normal acute response to inflammation and represent an adaptive response to an infection. The evolutionary significance (reflecting individual and social advantages) of this adaptative response likely explains why it is so highly conserved among species (Adelman and Martin, 2009; Miller and Raison, 2016).

It has been suggested that sickness behavior depends on the endotoxin-induced release of proinflammatory cytokines, the activation of microglia through Toll-like receptor (TLR) 4, the dysregulation of the kynurenine pathway, and the production of prooxidative species (Segreti et al., 1997; Lestage et al., 2002; Kelley et al., 2003; Hines et al., 2013; Eimerbrink et al., 2016).

LPS-induced changes in behavior have been largely attributed to the increased expression of proinflammatory cytokines (i.e., TNF-α, IL-1β, and IL-6).

Overall, the expression levels of these proinflammatory markers were lower, mainly in the dorsal part of the hippocampus, in saline-exposed animals that received a VTX pretreatment compared to controls. However, our results suggest that VTX pretreatment, rather than inhibiting the initial proinflammatory cascade and microglia activation, seems to increase the reactivity to the immune challenge in an area-dependent manner. An immunomodulatory role for VTX is supported by a main effect of diet on IL-1β expression levels in the ventral hippocampus. Moreover, *post hoc* analyses showed exclusively in VTX-fed animals a significant induction of IL-6 and TFN-α mRNAs in the dorsal and ventral parts of the hippocampus, respectively.

Indoleamine-2,3-dioxygenase (IDO), a tryptophan-degrading enzyme that metabolizes tryptophan into L-kynurenine (Stone and Darlington, 2002), is induced by proinflammatory cytokines and has been suggested to play a role in mediating LPS-induced depressive-like behavior (Pemberton et al., 1997; Takikawa et al., 1999; Fujigaki et al., 2006; Kwidzinski and Bechmann, 2007; O’Connor et al., 2009a). Again, IDO1 mRNA levels, as observed for proinflammatory cytokines, were lower in the dorsal hippocampus of saline-exposed VTX-pretreated animals compared to saline control animals. Preclinical models indicate that IDO is a key mediator of LPS-induced anhedonia in rodents (Salazar et al., 2012; Lawson et al., 2013). Here we found that a pretreatment with VTX was unable to prevent changes in sucrose consumption (anhedonia) induced by LPS.

During an immune challenge, proinflammatory cytokines can be both produced by microglia cells as well as affect their phenotype; we then measured expression levels of markers suggestive of microglia proinflammatory (M1) phenotype (Franco and Fernández-Suárez, 2015; Hu et al., 2015). In an oversimplified vision, microglia can be polarized into classical (M1) proinflammatory and alternative (M2) anti-inflammatory phenotype (Saijo and Glass, 2011; Hu et al., 2015; Stratoulias et al., 2019). The role of the diet in modulating LPS-induced effects was even more evident in the expression of surface microglia M1 markers than what was observed for proinflammatory cytokines. In fact, the chronic treatment with VTX decreased the expression of markers of microglial activation, such as CD86 and CD11b in the dorsal hippocampus. These results suggest that microglia may be a target of this antidepressant and further confirm the immunomodulatory activity of VTX (Talmon et al., 2018). Moreover, an interaction between diet and LPS treatment was demonstrated for all the CD molecules evaluated suggestive of M1 microglia phenotype in the dorsal hippocampus, while for CD86 and CD11b, this effect was present in the ventral hippocampus as well, indicating that VTX modulated microglia activation and reactivity to environmental stimuli like the well-known M1 polarization inducer LPS (Liu et al., 2012; Orihuela et al., 2016).

Besides regulating the expression of proinflammatory markers, LPS also regulates anti-inflammatory mediators, including anti-inflammatory cytokines (i.e., IL-4 and transforming growth factor or TGF-β1), cytokine antagonists (i.e., IL-1 receptor antagonist–IL-1Ra), and markers of M2 phenotype (i.e., CD206 and arginase-Arg-1) (Fenn et al., 2012; Chhor et al., 2013; Lively and Schlichter, 2018; Golia et al., 2019). This is important, for example, to limit the duration of the immune response through the brain and to prevent possible central immune-mediated damage. Enhancing M2 microglia polarization has been suggested as an important mechanism to inhibit neuroinflammation induced by LPS (Kobayashi et al., 2013; Yang et al., 2017; Bok et al., 2018; Liu et al., 2019; Zhang et al., 2019). Here, we demonstrated that, in animals fed VTX, the immune challenge strongly increased the expression of IL-4 both in the dorsal and in the ventral parts of the hippocampus. IL-4 is a known M2 polarization inducer (Liu et al., 2016). Recently, it has been demonstrated in rats that VTX (10 mg/kg, 10 days orally) increased central expression of IL-4 24 h after a LPS injection (Tomaz et al., 2020). Moreover, studies on isolated human monocytes (that share similarities with microglia) have shown that VTX drives monocytes/macrophages towards an anti-inflammatory phenotype (Talmon et al., 2018). Finally, in the dorsal hippocampus, an interaction between LPS and diet was observed in the expression levels of TGF-β1, with a smaller relative reduction, following LPS exposure, in VTX-exposed animals. As a whole, data on the expression of anti-inflammatory markers, including the IL-1 receptor antagonist (IL-1Ra) and markers of M2 activation, suggest that, at the same time point (6 h after LPS injection), VTX potentiated a negative regulation of the inflammatory response.

Consistent with clinical and preclinical findings showing that VTX impacts cognitive function, here, we demonstrated that VTX prevented the cognitive impairment induced by LPS (Katona et al., 2012; McIntyre et al., 2014; Li et al., 2015). It has been suggested that these effects depend on the inhibition of the immune response to LPS at central, hippocampal, level (Sorrenti et al., 2018). Increased synthesis and release of proinflammatory cytokines in the brain and activation of microglia are among the molecular and cellular contributors to the behavioral impairments induced by systemic LPS exposure. Cytokines are known to affect behavior, including memory and cognition. However, both immune activation and suppression affect hippocampal plasticity and impair memory performance (Yirmiya and Goshen, 2011). We recently contributed to a further demonstration that neural plasticity requires inflammatory responses to be kept within a tightly controlled range (Alboni et al., 2016; Golia et al., 2019).

Here, we demonstrated that LPS-induced effects on hippocampal memory could also be prevented without inhibiting the initial cytokines cascade that is necessary for mounting the adaptative response to infections. This could depend on a different balance between proinflammatory *vs.* anti-inflammatory activation or timing of activation of the anti-inflammatory response in VTX-exposed animals.

For example, the role of IL-1 in modulating the development of LPS- induced cognitive decline has been proposed (Terrando et al., 2010). Although IL-1β is critically involved in hippocampal plasticity and memory processes, high levels of this cytokine can lead to cognitive impairment (Rachal Pugh et al., 2001; Chen et al., 2008). We found that LPS increased the expression of IL-1β mRNA in the dorsal hippocampus, which is, mainly involved in memory processes, of both control and VTX-exposed animals (of about three- and fourfold increase with respect to saline, respectively). However, the increased expression of this cytokine takes place along with a stronger LPS-induction of the expression of the IL-1Ra, which acts by limiting IL-1β-mediated proinflammatory actions, in the same area (of about 58- and 67-fold increase, respectively, for control and VTX-exposed animals). IL-1β effects in the brain depend on the equilibrium between the levels of the cytokine and its negative regulator. Indeed, Terrando and colleagues found that a pretreatment with IL-1Ra significantly reduces microglia activation and cognitive dysfunction induced by LPS injection (Terrando et al., 2010).

It is also possible that this effect on memory may depend on the regulation of other targets different than cytokines and directly related molecules.

## Conclusion

The aim of the present study was to determine if a chronic pretreatment with VTX was able to prevent the behavioral alterations elicited by an LPS challenge by acting directly on the inflammatory cascade in the hippocampus. We showed that VTX selectively prevents the onset of an LPS-dependent cognitive impairment without interfering with the proinflammatory cascade that leads to the adaptive sickness response. Investigating the molecular mechanisms that underlie these events in key areas, like the hippocampus, but also the prefrontal cortex or the nucleus accumbens, will open new interesting perspectives for a better understanding of the role of neuroinflammation in neuronal plasticity and cognitive functions.

Moreover, a deeper insight into the mechanisms underlying cognitive dysfunctions in MDD could improve the clinical management of depression in a long-term perspective since cognitive symptoms not only represent a consistent social burden but are also those that tend to become chronic (Bortolato et al., 2014; Lam et al., 2014; Knight and Baune, 2018).

## Data Availability

The authors acknowledge that the data presented in this study must be deposited and made publicly available in an acceptable repository, prior to publication. Frontiers cannot accept an article that does not adhere to our open data policies.
