# Supplementary material for: Vortioxetine Prevents Lipopolysaccharide-Induced Memory Impairment Without Inhibiting the Initial Inflammatory Cascade
Source: Front Pharmacol. 2021 Feb 4;11:603979. doi: 10.3389/fphar.2020.603979 (PMC7890663; doi:10.3389/fphar.2020.603979)
Supplement: Supplementary file 1 [file datasheet1.pdf]

Supplementary Material

Supplementary Table 1: Nucleotide sequence of the forward and reverse primers used for Real-Time PCR.

Target name	Primer Sequence (5' -3')	Gene Bank Number
TNF-α	Fw GGCCTCCCTCTCATCAGTTC<br>Rv CACTTGGTGGTTTGCTACGA	NM_013693.3
IL-1β	Fw TGAAAGCTCTCCACCTCAATG<br>Rv CCAAGGCCACAGGTATTTTG	NM_008361.4
IL-6	Fw CTTCACAAGTCGGAGGCTTA<br>Rv CAAGTGCATCATCGTTGTTC	NM_031168.2
IDO1	Fw TGCAGACTGTGTCCTGGCAAACT<br>Rv GCCCTTGTCGCAGTCCCCAC	NM_008324.2
CD14	Fw AGATGTGGAATTGTACGGCG<br>Rv CGTAAGCCGCTTTAAGGACA	NM_009841.4
CD86	Fw GTAGACGTGTTCCAGAACTTA<br>Rv TGTTTTGAGCCTTTGTAAAT	NM_019388.3
CD11b	Fw ACGCCATCTACATGATTGTCAC<br>Rv AAGACTACACTGACAGGGAGGC	NM_001082960.1
IL-4	Fw CCAAGGTGCTTCGCATATTT<br>Rv ATCGAAAAGCCCGAAAGAGT	NM_021283.2
TGF-β1	Fw CAAGGGCTACCATGCCAACTT<br>Rv GTTGTGTTGGTTGTAGAGGGC	NM_011577.2
IL-1Ra	Fw AAGCCTTCAGAATCTGGGATAC<br>Rv TCATCTCCAGACTTGGCACA	NM_031167.5
CD206	Fw CAAGGAAGGTTGGCATTTGT<br>Rv CCTTTCAGTCCTTTGCAAGT	NM_008625.2
Arg1	Fw CTCCAAGCCAAAGTCCTTAGAG<br>Rv AGGAGCTGTCATTAGGGACATC	NM_007482.3
CypA	Fw AGCATACAGGTCCTGGCATC<br>Rv TTCACCTTCCCAAAGACCAC	NM_008907.2

Supplementary Table 2: Summary of the statistical analysis for the behavioural data

*Mice receiving standard or VTX enriched diet for 4 weeks received an intraperitoneal injection of either LPS or saline. Six hours after LPS or vehicle injection feeding, body weight, behaviour in the open field and serum corticosterone levels were evaluated, while 24 hours after the immune challenge we assessed behaviour in the open field, sucrose preference and performance in the novel object recognition test. Data were analyzed with a two-factor (diet x LPS) analysis of variance (ANOVA); * p<0.05; ** p<0.01, *** p<0.001*

Parameter			Univariate ANOVA	Main effects		Interaction
				LPS	DIET	LPS*DIET
**LPS effect (6 hours)**	***Body weight***		**<br>*p* =0.001<br>F(3;43)=6.402	***<br>*p* <0.0001<br>F(1;43)=18.683	↔<br>*p* =0.379<br>F(1;43)=0.541	↔<br>*p* =0.959<br>F(1;43)=0.003
	Food intake		↔<br>*p* =0.056<br>F(3;43)=2.729	*<br>*p* =0.013<br>F(1;43)=6.687	↔<br>*p* =0.348<br>F(1;43)=0.900	↔<br>*p* =0.467<br>F(1;43)=0.539
**Open field (6 hours after LPS)**	***Total distance (whole arena)***		***<br>*p* <0.0001<br>F(3;41)=26.850	***<br>*p* <0.0001<br>F(1;41)=62.805	↔<br>*p* =0.074<br>F(1;41)=3.415	↔<br>*p* =0.114<br>F(1;41)=2.642
	Rearings		***<br>*p* <0.0001<br>F(3;41)=18.284	***<br>*p* <0.0001<br>F(1;41)=47.596	↔<br>*p* =0.786<br>F(1;41)=0.087	↔<br>*p* =0.092<br>F(1;41)=2.975
	Total resting time		***<br>*p* <0.0001<br>F(3;43)=14.121	***<br>*p* <0.0001<br>F(1;43)=34.124	↔<br>*p* =0.490<br>F(1;43)=0.489	↔<br>*p* =0.443<br>F(1;43)=0.606
	Percent Time in the center		*<br>*p* =0.033<br>F(3;41)=3.207	↔<br>*p* =0.077<br>F(1;41)=3.330	↔<br>*p* =0.179<br>F(1;41)=1.884	↔<br>*p* =0.666<br>F(1;41)=0.189
Serum corticosterone			***<br>*p* <0.0001<br>F(3;42)=51.098	***<br>*p* <0.0001<br>F(1;42)=153.023	↔<br>*p* =0.810<br>F(1;42)=0.056	↔<br>*p* =0.322<br>F(1;42)=1.000
**SPT**	***Sucrose preference 24 hours***		**<br>*p* =0.010<br>F(3;37)=4.479	***<br>*p* =0.001<br>F(1;37)=12.718	↔<br>*p* =0.351<br>F(1;37)=0.896	↔<br>*p* =0.636<br>F(1;37)=0.228
	Liquid intake 24 hours		↔<br>*p* =0.397<br>F(3;43)=1.013	↔<br>*p* =0.993<br>F(1;43)=0.000	↔<br>*p* =0.162<br>F(1;43)=2.030	↔<br>*p* =0.322<br>F(1;43)=1.005
**Open field (24 hours after LPS)**	***Total distance (whole arena)***		↔<br>*p* =0.651<br>F(3;43)=0.550	↔<br>*p* =0.961<br>F(1;43)=0.002	↔<br>*p* =0.112<br>F(1;43)=2.672	↔<br>*p* =0.998<br>F(1;43)=0.000
	Rearings		↔<br>*p* =0.191<br>F(3;41)=1.656	↔<br>*p* =0.198<br>F(1;41)=1.712	↔<br>*p* =0.164<br>F(1;41)=2.013	↔<br>*p* =0.887<br>F(1;41)=0.024
	Total resting time		↔<br>*p* =0.954<br>F(3;43)=0.110	↔<br>*p* =0.843<br>F(1;43)=0.40	↔<br>*p* =0.741<br>F(1;43)=0.111	↔<br>*p* =0.678<br>F(1;43)=0.117
	Percent Time in the center		↔<br>*p* =0.323<br>F(3;43)=1.198	↔<br>*p* =0.172<br>F(1;43)=1.946	↔<br>*p* =0.776<br>F(1;43)=0.082	↔<br>*p* =0.207<br>F(1;43)=1.653
**NORT**	**Training**	***Total interaction***	↔<br>*p* =0.839<br>F(3;43)=0.280	↔<br>*p* =0.743<br>F(1;43)=0.109	↔<br>*p* =0.829<br>F(1;43)=0.050	↔<br>*p* =0.409<br>F(1;43)=0.697
		Total distance travelled	↔<br>*p* =0.120<br>F(3;43)=2.067	↔<br>*p* =0.179<br>F(1;43)=1.870	↔<br>*p* =0.204<br>F(1;43)=1.670	↔<br>*p* =0.111<br>F(1;43)=2.059
		Total exploring time	↔<br>*p* =0.256<br>F(3;43)=1.401	↔<br>*p* =0.190<br>F(1;44)=1.774	↔<br>*p* =0.929<br>F(1;44)=0.008	↔<br>*p* =0.137<br>F(1;44)=2.299
	**Test**	***Total interaction***	↔<br>*p* =0.340<br>F(3;43)=1.151	↔<br>*p* =0.090<br>F(1;43)=3.014	↔<br>*p* =0.189<br>F(1;43)=0.666	↔<br>*p* =0.550<br>F(1;43)=0.363
		Total distance travelled	↔<br>*p* =0.275<br>F(3;43)=1.338	↔<br>*p* =0.140<br>F(1;43)=2.262	↔<br>*p* =0.198<br>F(1;43)=1.711	↔<br>*p* =0.679<br>F(1;43)=0.173
		Total exploring time	↔<br>*p* =0.624<br>F(3;43)=0.586	↔<br>*p* =0.203<br>F(1;44)=1.670	↔<br>*p* =0.859<br>F(1;44)=0.032	↔<br>*p* =0.850<br>F(1;44)=0.036

Supplementary Table 3: Summary of statistical analysis for gene expression results in the dorsal and ventral hippocampus in animals chronically fed with standard diet or VTX-enriched diet sacrificed 6 hours after receiving LPS or saline.

*Expression levels of mRNAs coding for pro-inflammatory cytokines (TNF-α, IL-1β and IL-6), IDO1, markers of activated microglia (CD14, CD86 and CD11b), anti-inflammatory cytokines (IL-4 and TGF-β1), cytokine antagonist (IL-1Ra) or markers of microglia M2 phenotype (CD206 and Arg1) in the dorsal hippocampus or in the ventral hippocampus were evaluated using Real Time PCR. Mice receiving standard or VTX enriched diet for 4 weeks were sacrificed 6 hours after an intraperitoneal injection of either LPS or saline. The mRNA levels were normalized to the endogenous control, cyclophilin A. For a quantitative evaluation of change comparative ΔΔCt method was performed using as calibrator, average of saline control animals. Data were analyzed with a two-factor (diet x LPS) analysis of variance (ANOVA); * p<0.05; ** p<0.01, *** p<0.001*

TARGETS	*Dorsal hippocampus*				*Ventral hippocampus*			
	Univariate ANOVA	Main effects		Interaction	Univariate ANOVA	Main effects		Interaction
		LPS	DIET	LPS*DIET		LPS	DIET	LPS*DIET
TNF-a	***<br>p <0.0001<br>F(3;36)=32.403	***<br>p <0.0001<br>F(1;36)=96.822	↔<br>p =0.417<br>F(1;36)=0.674	↔<br>p =0.244<br>F(1;36)=1.407	***<br>p <0.0001<br>F(3;37)=30.529	***<br>p <0.0001<br>F(1;37)=88.535	↔<br>p =0.202<br>F(1;37)=1.690	↔<br>p =0.106<br>F(1;37)=2.757
IL-1β	***<br>p <0.0001<br>F(3;35)=21.581	***<br>p <0.0001<br>F(1;35)=43.759	↔<br>p =0.382<br>F(1;35)=0.785	↔<br>p =0.886<br>F(1;35)=0.021	***<br>p <0.0001<br>F(3;36)=11.637	***<br>p <0.0001<br>F(1;36)=23.375	**<br>p =0.005<br>F(1;36)=9.119	↔<br>p =0.094<br>F(1;36)=2.973
IL-6	**<br>p =0.004<br>F(3;36)=5.242	***<br>p =0.001<br>F(1;36)=14.046	↔<br>p =0.695<br>F(1;36)=0.157	↔<br>p =0.250<br>F(1;36)=1.373	**<br>p =0.004<br>F(3;37)=5.296	***<br>p =0.001<br>F(1;37)=13.547	↔<br>p =0.299<br>F(1;37)=1.111	↔<br>p =0.133<br>F(1;37)=2.367
IDO1	***<br>p <0.0001<br>F(3;36)=16.250	***<br>p <0.0001<br>F(1;36)=33.931	↔<br>p =0.212<br>F(1;36)=1.623	***<br>p =0.001<br>F(1;36)=12.669	***<br>p <0.0001<br>F(3;36)=15.803	***<br>p <0.0001<br>F(1;36)=42.075	↔<br>p =0.182<br>F(1;36)=1.862	↔<br>p =0.062<br>F(1;36)=3.742
CD14	***<br>p <0.0001<br>F(3;37)=23.949	***<br>p <0.0001<br>F(1;37)=61.357	↔<br>p =0.053<br>F(1;37)=4.030	*<br>p =0.012<br>F(1;37)=6.991	***<br>p <0.0001<br>F(3;36)=25.943	***<br>p <0.0001<br>F(1;36)=75.758	↔<br>p =0.108<br>F(1;36)=2.726	↔<br>p =0.245<br>F(1;36)=1.403
CD86	***<br>p <0.0001<br>F(3;37)=8.023	***<br>p =0.001<br>F(1;37)=14.777	↔<br>p =0.206<br>F(1;37)=1.661	*<br>p =0.011<br>F(1;37)=7.240	***<br>p <0.0001<br>F(3;37)=13.414	***<br>p <0.0001<br>F(1;37)=27.468	**<br>p =0.005<br>F(1;37)=8.862	**<br>p =0.008<br>F(1;37)=7.958
CD11b	**<br>p =0.001<br>F(3;38)=7.352	↔<br>p =0.395<br>F(1;38)=0.743	**<br>p =0.004<br>F(1;38)=9.296	**<br>p =0.002<br>F(1;38)=11.456	**<br>p =0.007<br>F(3;36)=4.858	↔<br>p =0.095<br>F(1;36)=2.957	*<br>p =0.038<br>F(1;36)=4.682	*<br>p =0.012<br>F(1;36)=7.047
IL-4	***<br>p <0.0001<br>F(3;35)=15.915	***<br>p <0.0001<br>F(1;35)=32.073	**<br>p =0.003<br>F(1;35)=10.171	**<br>p =0.005<br>F(1;35)=9.244	***<br>p <0.0001<br>F(3;35)=19.079	***<br>p <0.0001<br>F(1;35)=31.216	***<br>p <0.0001<br>F(1;35)=21.087	**<br>p =0.006<br>F(1;35)=8.493
TGF- β1	***<br>p <0.0001<br>F(3;36)=21.690	***<br>p <0.0001<br>F(1;36)=54.109	↔<br>p =0.990<br>F(1;36)=0	**<br>p =0.004<br>F(1;36)=9.674	***<br>p <0.0001<br>F(3;37)=27.557	***<br>p <0.0001<br>F(1;37)=79.776	↔<br>p =0.351<br>F(1;37)=0.895	↔<br>p =0.182<br>F(1;37)=1.855
IL-1Ra	***<br>p <0.0001<br>F(3;35)=27.511	***<br>p <0.0001<br>F(1;35)=81.799	↔<br>p =0.346<br>F(1;35)=0.561	↔<br>p =0.387<br>F(1;35)=0.538	***<br>p <0.0001<br>F(3;35)=8.887	***<br>p <0.0001<br>F(1;35)=28.880	↔<br>p =0.160<br>F(1;35)=2.065	↔<br>p =0.155<br>F(1;35)=2.126
CD206	**<br>p =0.001<br>F(3;37)=7.364	***<br>p <0.0001<br>F(1;37)=19.486	↔<br>p =0.431<br>F(1;37)=0.636	↔<br>p =0.223<br>F(1;37)=1.543	***<br>p <0.0001<br>F(3;35)=11.953	***<br>p <0.0001<br>F(1;35)= 35.76	↔<br>p =0.809<br>F(1;35)=0.059	↔<br>p =0.841<br>F(1;35)=0.041
Arg1	↔<br>p =0.327<br>F(3;37)=1.192	↔<br>p =0.944<br>F(1;37)=0.005	↔<br>p =0.610<br>F(1;37)=0.265	↔<br>p =0.078<br>F(1;37)=3.309	↔<br>p =0.380<br>F(3;37)=1.057	↔<br>p =0.308<br>F(1;37)=1.072	↔<br>p =0.581<br>F(1;37)=0.311	↔<br>p =0.183<br>F(1;37)=1.845

Supplementary result: food and water consumption during a 28-day dietary administration of vortioxetine or standard diet

During the experimental period we monitored food and water consumption. No gross changes in animal's feeding and drinking behavior were found. Fluctuations were observed in food intake at day 11, where VORT-receiving animals ate more than CTRL (t=-2.467; p=0.022), and at day 28, when the opposite happened (t=2.510; p=0.031). As for water consumption, there were no significant differences between groups during the course of the experiment. On the 29th day, before receiving either LPS or saline, no difference was found in food and water consumption between mice treated chronically with vortioxetine and those fed with standard diet.

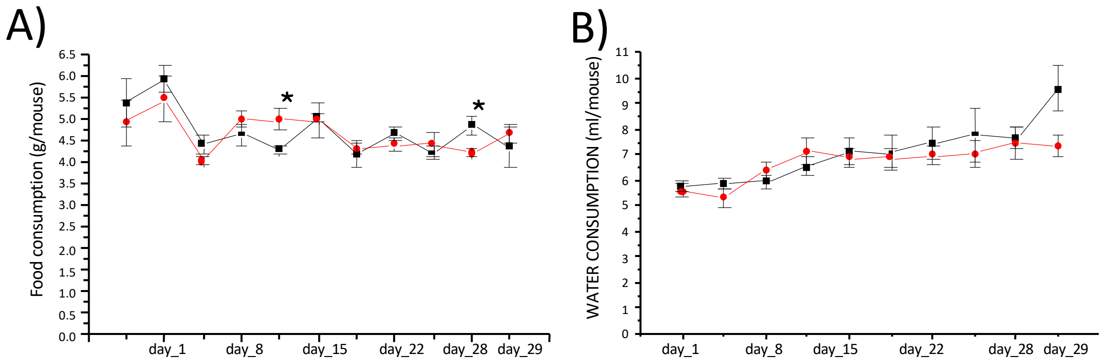

Supplementary figure 1: Food intake (A) and water consumption (B) in male C57BL6/J mice receiving either standard diet or chronic dietary administration of vortioxetine for 4 weeks. n= 43-44 mice per group. Data are represented as means ± S.E.M. and were analysed with t-test (standard diet ■ *vs* VTX-enriched diet ●) *p < 0.05 vs standard diet.

Supplementary figure 2

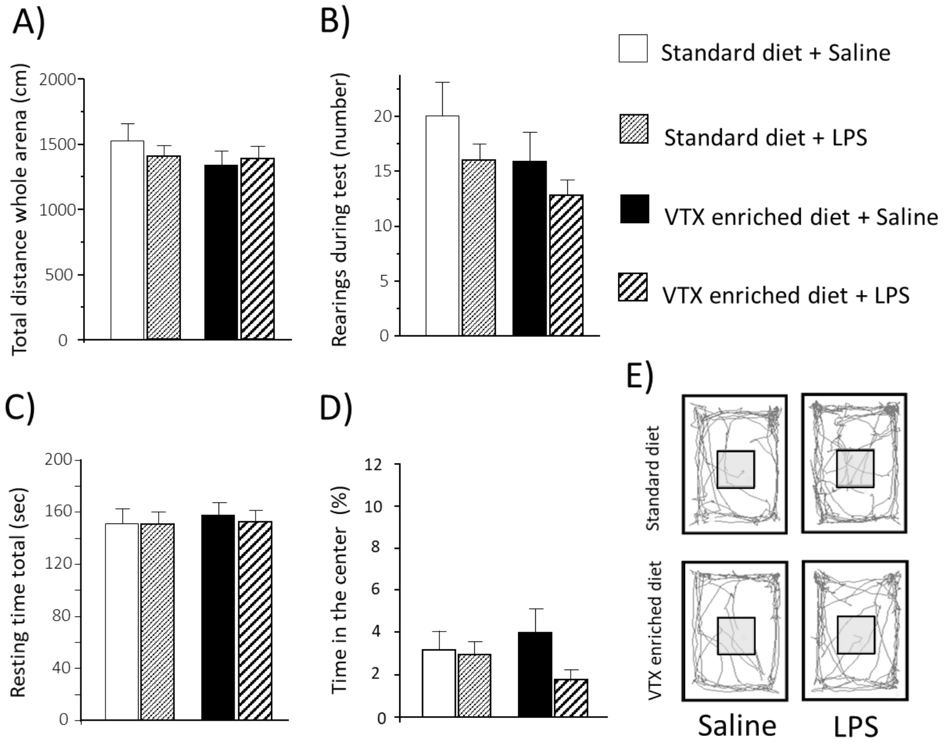

Supplementary figure 2: 24 hours after LPS/saline injection, animals did not display any alteration in locomotor activity or anxiety-like behavior, irrespective of the diet they received before the immune challenge. Histograms represent the behaviors in the open field test. (A) Total distance travelled (cm) in the entire open field area. (B) Number of rearing during test. (C) Resting time during test. (D) Percent time spent in the center of the open field. (E) Representative movement traces of all groups in the open field. Treatment as indicated in the legend, n= 12 mice per group. Data are represented as means ± S.E.M.
